# Identification of a Small Molecule That Selectively Inhibits Mouse PC2 over Mouse PC1/3: A Computational and Experimental Study

**DOI:** 10.1371/journal.pone.0056957

**Published:** 2013-02-22

**Authors:** Austin B. Yongye, Mirella Vivoli, Iris Lindberg, Jon R. Appel, Richard A. Houghten, Karina Martinez-Mayorga

**Affiliations:** 1 Torrey Pines Institute for Molecular Studies, Port St Lucie, Florida, United States of America; 2 Department of Anatomy and Neurobiology, University of Maryland School of Medicine, Baltimore, Maryland, United States of America; 3 Torrey Pines Institute for Molecular Studies, San Diego, California, United States of America; 4 Instituto de Química, UNAM, Mexico City, Mexico; Universite de Sherbrooke, Canada

## Abstract

The calcium-dependent serine endoproteases prohormone convertase 1/3 (PC1/3) and prohormone convertase 2 (PC2) play important roles in the homeostatic regulation of blood glucose levels, hence implicated in diabetes mellitus. Specifically, the absence of PC2 has been associated with chronic hypoglycemia. Since there is a reasonably good conservation of the catalytic domain between species translation of inhibitory effects is likely. In fact, similar results have been found using both mouse and human recombinant enzymes. Here, we employed computational structure-based approaches to screen 14,400 compounds from the Maybridge small molecule library towards mouse PC2. Our most remarkable finding was the identification of a potent and selective PC2 inhibitor. Kinetic data showed the compound to be an allosteric inhibitor. The compound identified is one of the few reported selective, small-molecule inhibitors of PC2. In addition, this new PC2 inhibitor is structurally different and of smaller size than those reported previously. This is advantageous for future studies where structural analogues can be built upon.

## Introduction

Pro-protein convertases (PC) belong to the class of calcium-dependent serine endoproteases responsible for the conversion of inactive protein precursors (peptide hormones, enzymes, receptors, growth factors, neuropeptides, etc) to their active forms [1], [2]. Currently, seven mammalian PCs have been identified: furin, PC1/3, PC2, PC4, PACE4, PC5/6 and PC7/PC8. The corresponding genes encoding these enzymes in humans are *FURIN, PCSK1, PCSK2, PCSK4, PCSK5, PCSK6* and *PCSK7*. [3] These PCs cleave their substrates C-terminal to a basic amino acid residue by recognizing the R-*X*-K/R-R↓ multibasic motif, typically after an arginine residue [1], where *X* can be any amino acid except cysteine, and the arrow denotes the site of cleavage. These enzymes are involved in key processes such as embryogenesis [4] and blood sugar homeostasis [5].

PC1/3 and PC2 are expressed primarily in neuroendocrine tissues [6], [7], [8], and are well conserved between rodents and humans [9], [10], [11] PC1/3 and PC2 function in conjunction with carboxypeptidase E (CPE) in the central nervous system to generate active endogenous opioid and other neuropeptides from their precursors [12]. In pancreatic α-cells PC2 and CPE extract active glucagon from pro-glucagon [8], while in pancreatic β-cells both PC1/3 and PC2 (along with CPE) act synergistically to excise insulin from pro-insulin [8]. Nonetheless, PC1/3 is the primary converter of pro-insulin to insulin [13]. In intestinal L cells, PC1/3 extracts two peptides from proglucagon, glucagon-like peptides (GLP) 1 and 2. GLP1_7−37_ also up-regulates insulin secretion from β-cells in response to high glucose levels [8]. Thus PC1/3 and PC2 play crucial roles in the homeostatic regulation of plasma glucose levels. In agreement with this idea, chronic hypoglycemia has been observed in PC2 double knock-out mice due to defects in processing proglucagon [8]. However, a deficiency in functioning PC1/3 has been cited as a major cause for severe obesity in human subjects [2], [14], [15] as well as in many human populations [15], [16]. Consequently, selective inhibition of PC2 over PC1/3 is expected to be crucial in the treatment of chronic hyperglycemia (diabetes mellitus) using PC2-directed drugs. Conversely, selective PC1/3 inhibitors may be useful in instances of a neuroendocrine-related cancer such as insulinoma. It can be speculated that PC modulators with low toxicity and acceptable ADMET properties will be effective therapies for the treatment for these diseases.

Four different strategies, discussed in the succeeding paragraphs, have been explored in the search for selective PC1/3 and PC2 inhibitors: 1) endogenous peptide inhibitors found in the secretory pathway [10], [17], [18], [19]; 2) development and testing of pro-domains [20], [21] and oligopeptides (from synthetic peptide combinatorial libraries) containing the primary activation cleavage motif [22], [23]; 3) peptidomimetics based on the cleavage activation motif; and 4) non-peptidyl small molecules [24], [25]. Of note, similar results have been found using both mouse and human recombinant enzymes, most likely because of the good conservation in the catalytic domain. The amino acid homologies between the catalytic domains of rat, human, and mouse PC1/3, PC2 and furin are in the range of 51 to 68% conservation [9]. Because of this reasonably good conservation of the catalytic domain translation of inhibitory effects between species is likely.

The N-terminal domain of the endogenous neuroendocrine protein 7B2 is required for the generation of active PC2 from proPC2 [26], and assists its transport through the secretory pathway [19]. However, its carboxy terminus is a selective, potent inhibitor of PC2 and does not inhibit PC1/3 [18]. Furthermore, the cystatin-related epididymal spermatogenic (CRES) protein is a selective inhibitor of PC2 over PC1/3 [6]. On the other hand, the neuroendocrine precursor proSAAS selectively inhibits PC1/3 over PC2, and is active in the high nM range [27]. This selective inhibition by proSAAS has been attributed to a hexapeptide sequence in its C-terminus [17].

In investigations employing the pro-domains of PC1/3 [20] and PC2 [21], the pro-peptides displayed low nanomolar, slow tight-binding inhibition of their respective activated enzymes. However, some chimeric PCs may exhibit cross-inhibition [26]. For example, when the propeptide of the PC1/3 protein is substituted with that of furin the enzyme retained activity. In contrast, when the propeptides of PC1/3 and PC2 were exchanged, the resultant mutants completely lost proteolytic activity [28]. Lack of selectivity, coupled with the expression of multiple PCs within the same cell, has been suggested as one potential drawback in employing pro-domains as inhibitors [26], especially when selectivity is required. For example, the proPC1/3 propeptide is an inhibitor of both PC1/3 and furin [20]. The large size of the PC propeptide, about 10 kDa, is another disadvantage to the use of prodomains as therapeutic inhibitors.

In the case of oligopeptides derived from the primary cleavage site, greater inhibition has generally been observed towards PC1/3 as compared to PC2 [22]. The weaker inhibition of PC2 is exemplified by a synthetic peptide combinatorial library of hexapeptides derived from the C-terminal of the pro-domain or primary cleavage site of PC2: low percent inhibition (≤54%) and Ki (sub-micromolar) values were observed for PC2 inhibition; on the other hand, peptides derived from the C-terminal pro-domain of PC1/3 were nM inhibitors of PC1/3, and showed higher percent inhibition values (≤75%) [18]. Interestingly, another hexapeptide (Ac-NVVAKK-NH_2_) that was included in the deconvoluted peptide library because of its presence within the PC2 binding site of the C-terminus of the endogenous PC2 inhibitor, 7B2, showed no inhibition of PC2 [22], and later studies showed that a full 14 residues of this peptide is required for potent inhibition [10]. Other studies have incorporated active site-directed groups such as chloromethylketones into peptide inhibitors of PC1/3 [29]. However, these irreversible inhibitors are likely to exhibit cytotoxic properties due to the presence of the chloromethylketone moiety, a known toxic agent.

Recently, a number of synthetic peptidomimetics were reported which exhibited great potency and selectivity towards PC1/3 over PC2 [30]. While non-peptidyl inhibitors have been previously reported against PC1/3 [31] only recently has a group of small-molecule inhibitors, pyrrolidine bis-piperazines, been identified for PC2 [24]. Subsequently, four competitive inhibitors of PC1/3 were identified from a series of compounds containing a 2,5-dideoxystreptamine scaffold [32]. In the same study three inhibitors of PC2 were identified which possess competitive, non-competitive and mixed inhibition mechanisms [32]. Given the proteolytic instabilities of peptides *in vivo*, and the relative dearth of small molecule inhibitors of PC1/3 and PC2, we decided to focus our attention on non-peptidyl small molecules.

Combinatorial libraries at the Torrey Pines Institute for Molecular Studies have provided potent ligands for different biological systems [33], [34], [35]. In the case of prohormone convertases, by using TPIMS combinatorial libraries the first small molecule inhibitors for PC2 were identified [24], as well as the naturally occurring peptide inhibitor for PC1/3 [17], [22], [27]. Thus, screening of combinatorial libraries has proven effective for the identification of PC inhibitors. To search for ligands with biological relevance, we have previously reported using a combination of mixture-based combinatorial library screening with virtual screening [36], [37]. In addition, we have performed molecular modeling studies of prohormone convertases to rationalize structure-activity relationships and to suggest alternative binding pockets for non-competitive inhibitors [32], [38]. In the work described below, we have explored the Maybridge data set as a source of PC inhibitors, based on the idea that molecules from this set would complement the chemical space covered by our previously reported inhibitors derived from small molecule and peptide combinatorial libraries. In particular, we have here employed molecular modeling followed by biological evaluation in an effort to identify potential selective, non-peptidyl small-molecule inhibitors of PC2 over PC1/3. The strategy employed is as follows: 1) Generate and validate homology models of mouse PC1/3 and PC2. The homology models were validated by assessing their ability to highly rank active compounds amongst a set of decoys (for PC2) or in the absence of decoys when there was a significant spread in the reported activities of ligands (for PC1/3); 2) Perform molecular docking of compounds procured from the Maybridge Hitfinder Collection® database, utilizing Glide version 5.5 [39] followed by FRED [40], employing the top-scoring compounds from Glide extended precision (XP) scoring; and 3) Propose potential selective compounds for biological screening.

## Results

Only recently have small molecule inhibitors with selectivity towards PC2 been reported with Ki values ranging from 540–660 nM [24]. We here present the results of our computational strategies to identify selective inhibitors of PC2 over PC1/3 and the corresponding experimental data.

### 1). Generating a Homology Model of Mouse PC2

The percent identities between the target and template sequences are shown in **Table 1**. The overall identities were significantly higher for furin (52%) compared to Kex2 (39%). The domain-specific identities were also higher for furin relative to Kex2: 56% and 44% for the catalytic domain; and 44% and 29% for the P-domain. Moreover, the alignments also generated gaps between the template and target sequences, though the percentage was slightly less for furin. As such, furin was designated as the primary source of Cartesian coordinates for the homology model. The process of using multiple templates necessitated the superposition of the templates, resulting in a 0.916-Å RMSD between 395 backbone atom pairs. Superpositions and sequence alignments of the homology model and templates are shown in **Figure S2**. A PROCHECK analysis of 411 non-proline and non-glycine residues is shown in **Table S1**. The distributions were as follows: most favored region, 83.7%; additional allowed region, 13.9%; generously allowed region, 1.5% and disallowed region 1.0% (representing four residues). Nonetheless, for docking purposes none of the outliers occurred in the binding pocket of the homology model, while 99% of the residues were in the generously allowed to the most favored regions. Therefore, this model was employed for the current docking studies.

**Table 1:** Selected peptides and small molecules active towards PC2.

Ligand	Type of inhibition	PC2 K_i_ (µM)	Reference
Ac-LLRVKR-NH_2_	competitive	0.360±0.50	Apletalina E. *et al.,* J. Biol. Chem. 273 (41), 26589, 1998
Ac-LMRVKR-NH_2_		0.530±0.70	
Ac-LKRVKR-NH_2_		0.620±0.150	
Ac-LYRVKR-NH_2_		0.720±0.50	
Ac-IIRVKR-NH_2_		0.530±0.120	
LLRVKR-NH_2_	competitive	2.3±0. 2	Cameron A. *et al.,* J. Biol. Chem. 275(47), 36741, 2000
Ac-LLRVKR	competitive	1.3±0.6	
369068 (1435-6)	noncompetitive	0.66±0.10	Kowalska D. *et al.,* Mol. Pharmacol. 75(3), 51334, 2009
92246 (1435-16)	noncompetitive	0.56±0.07	
92248 (1435-18)	noncompetitive	0.54±0.10	
369092 (1435-10)	noncompetitive	0.59±0.08	

### 2). Generating Structural Models of PC2 for Ligand Docking

A molecular dynamics (MD) simulation was performed employing the homology model in order to select several receptor conformations to account for receptor flexibility during docking. Seven structures, including the homology model and six from the MD simulation (*model1*, *model2*, *model3*, *model4*, *model5* and *model6*), were utilized. Details on how these structures were selected are provided in the Methods (section 3: Generating structural models of PC2 for ligand docking). To assess the ability of each model to rank active compounds highly in the docking procedure, a plot of the fraction of actives retrieved versus the fraction of the database screened was generated.

In drug discovery projects wherein databases generally consist of 10^4^–10^6^ compounds, it is computationally inefficient to scan the ranked docking scores for the entire database to retrieve active compounds. Typically, only the top 10% of docked compounds are analyzed. Thus, the recoveries and areas under curves (AUC) were determined only for the top 10% of the docked ligands, **Figure S3**. At this cut-off the performances were as follows: *EnsDock* (0.063), *model1* (0.057), *Homology* (0.054) = *model6* (0.054), *model5* (0.053), *model3* (0.051), and *model2* (0.050) = *model4* (0.050). The higher the AUC the more efficient a model was in distinguishing between the actives and decoys. Further analysis of the docked ligands in the top 10% of the rankings indicated that an ensemble composed from *Homology* and *model6* retrieved all seven peptides. Thus this ensemble was employed for further screening of 14,400 compounds downloaded from the Maybridge database.

### 3). Ligands and Decoys

Box plots comparing the distributions of the seven PK properties of the actives and their selected decoys are shown in **Figure S4**. Overall, there were overlaps in the property spaces of the acceptors, donors, logS, TPSA and SlogP descriptors. However, there were little and no overlaps for the molecular weights and rotatable bond descriptors, respectively, which can be attributed to the larger sizes of the peptides and pyrrolidine bis-piperazines employed as actives (**Figure S4**).

### 4). Docking of the Maybridge Database

Preparing the 14,400 Maybridge compounds resulted in 35,500 different tautomers and ionization states. Three docking stages were employed for each protein model: 1) a rigid high-throughput virtual screening stage; 2) next, the top 16,000 scoring compounds were selected for docking using Glide Standard Precision (GlideSP); and finally 3) the top 1600 compounds were passed onto the Glide Extended Precision (GlideXP) docking stage. The outputs from both protein models were merged and the top 500 compounds were analyzed. For cases in which different ionization states for the same compound were present among this set, their docking scores were averaged. This averaging resulted in 115 unique ligands.

**Figure 1:**
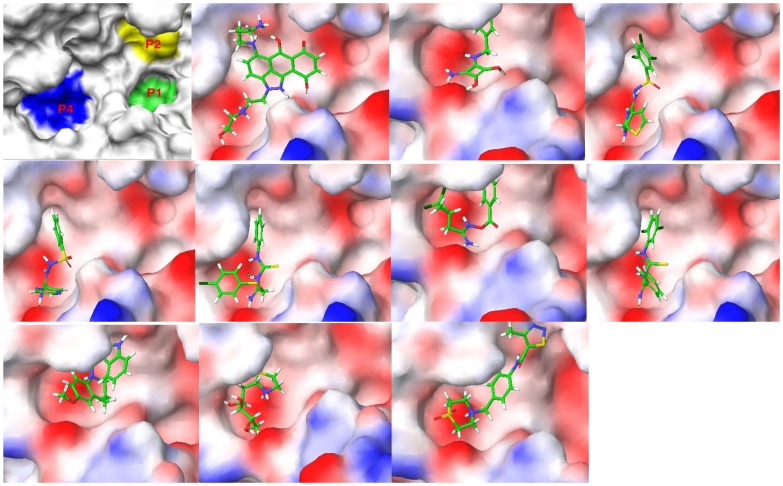
The binding surface of a homology model of mouse PC2 along with the top ten poses of the Maybridge database docked to PC2. In the first image the P1, P2 and P4 subsites with high electronegative potential are depicted in color, while in the subsequent images the surface of the enzyme is color-coded based on the electrostatic potential of the residues.

**Figure 2:**
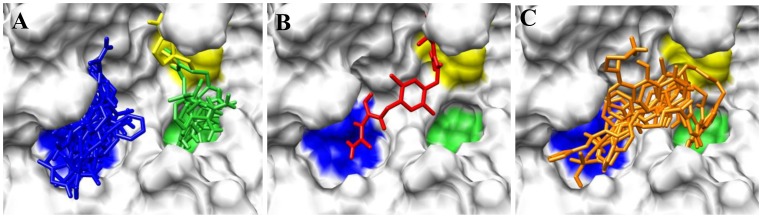
Distribution of ligands within subsites in the binding pocket of PC2. (A) Ligands accommodated in distinct subsites; (B) A ligand that spread into the P2 and P4 subsites; (C) Ligands that overlapped with the P1 and P4 sites, which may be used as frameworks to link fragments in the P1, P2 and P4 subsites shown in (A).

**Figure 3:**
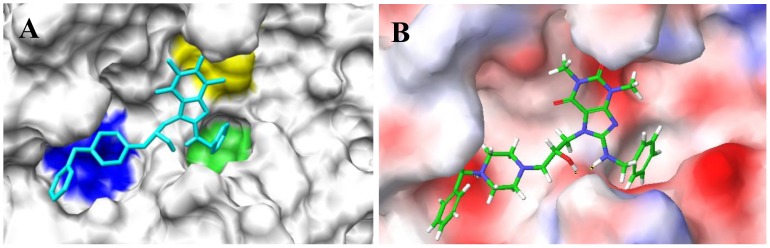
A Maybridge ligand that simultaneously occupies the P1, P2 and P4 sites in PC2. Generic surface plot, with the ligand occupying the three sites (A). The enzyme’s surface is rendered based on electrostatic potential (B). Rank, #55; Average docking score, −9.39; Weight = 517.634 Da.

**Figure 4:**
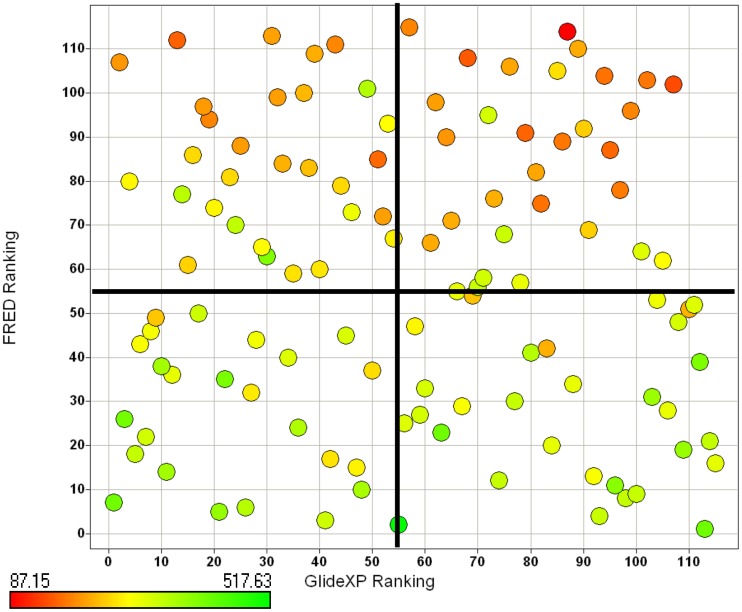
Distribution of molecular weights for 115 compounds docked utilizing both FRED and GlideXP. These are the top 115 scoring compounds obtained from docking the Maybridge database to the mouse PC2 models. The compounds are color-coded based on molecular weight. Yellow is the median and represents a molecular weight of 283.31 Da.

The binding poses of the top ten ranked Maybridge compounds and the binding pocket of the PC2 model are shown in **Figure 1**. The surface in the first panel is colored to indicate electronegative subsites close to the scissile bond, which are typically utilized by these enzymes for efficient substrate processing [7], [22]. As can be seen, the ligands docked predominantly in the P4 subsite. Notably, these compounds contained protonated or positively ionizable nitrogen atoms that formed salt bridges or hydrogen bonds with residues in this pocket, for example: the carboxyl groups of Glu139 and Asp167; the backbone carbonyl groups of Gly158, Met1278 and Thr135; and the hydroxyl oxygen atom of Tyr210. This preference may be driven by these interactions and an overall better fit at the P4 subsite by these ligands. However, viewing all the top 115 compounds in the binding pocket showed a distribution of the ligands within other subsites, **Figure 2**. **Figure 2A** shows ligands distinctly docked to the P1 (green), P2 (yellow) and P4 (blue) subsites. It can be seen that one ligand occupied both the P2 and P4 subsites, **Figure 2B**, while eight ligands overlapped with the P1 and P4 subsites, **Figure 2C**. One ligand, ranked #55 with an average Glide XP docking score of −9.39 kcal/mol, occupied all three pockets, **Figure 3**. Also worth noting were the small sizes of these top-ranked compounds. Hence, the FRED program that gives a higher weight to shape complementarity was utilized to re-dock the top 115 compounds. The rankings from FRED and GlideXP were compared to determine the extent to which FRED scored higher molecular weight compounds from this set more favorably than GlideXP, **Figure 4**. The compounds are color-coded based on molecular weights: red and green signify low and higher molecular weights, respectively. The thicker horizontal and vertical lines indicate approximately the halfway marks of the FRED and GlideXP rankings, respectively. From this plot, GlideXP scored both low and high molecular weight compounds in the top and bottom halves of the ranking. However, FRED scored the majority of high molecular compounds in the top half of the ranking, and vice versa for low molecular weight compounds. Therefore, the rankings from FRED and those from GlideXP were combined, to obtain a final consensus score.

### 5). Biological Assays

**Figure 5:**
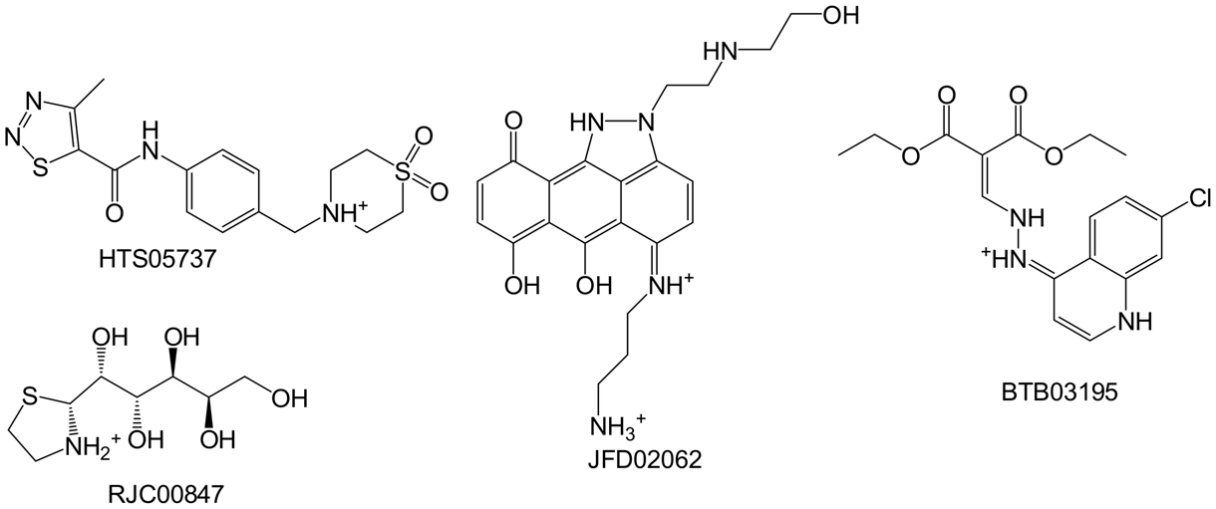
Compounds that activated PC2 (HTS05737 and JFD02062) and PC1/3 (BTB03195). RJC00847 selectively inhibited PC2.

**Figure 6:**
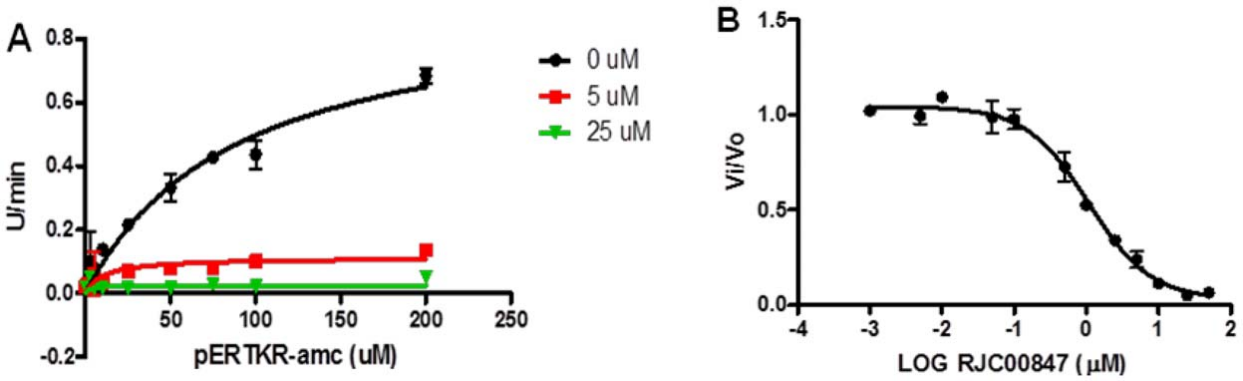
Enzymatic assay of RJC00847 against PC2. **A**). The effect of increasing the concentration of the inhibitor on the detection of fluorescent product, 7-amino-4-methylcoumarin (AMC). **B**). Concentration-response curve, from which an IC_50_ value of 1.1±0.06 µM was determined.

The percent inhibition of the compounds proposed for biological screening against PC1/3 and PC2 are shown in **Table 2**. While there was general selectivity towards PC2, as indicated by the higher inhibition of PC2 over PC1/3, some of the compounds stimulated the enzymes. For example HTS05737 showed a drastic activation (282%) of PC2 at 50 µM, while JFD02062 increased the activity of PC2 by 30% and 60% at 25 and 50 µM, respectively. The structures of these compounds are shown in **Figure 5**, HTS0537 and JFD02062 were found to be weak inhibitors of PC1/3. On the other hand, BTB03195 activated PC1/3, while inhibiting PC2 at both concentrations. One compound, RJC00847 showed percent inhibitions of PC2 of 98% and 86% at 50 and 25 µM, respectively, but low inhibitions of PC1/3 with values of 6% and 10% at 50 and 25 µM, respectively. RJC00847 was tested further at 5 µM. The Ki and IC_50_ values were 0.69±0.08 and 1.1±0.06 µM, respectively. The inhibitory plots of the enzymatic assays are shown in **Figure 6**. Unexpectedly, RJC00847 was found to be a non-competitive inhibitor of PC2, implying that it binds to an allosteric site on the enzyme, and represented a case in which the predicted and experimental poses were markedly different. This scenario is not unprecedented; an example is AmpC β-lactamase wherein a co-crystallized inhibitor bound to a site that was 16 Å away from the active site employed during docking [41]. This is the third study that has successfully identified a selective, non-peptide inhibitor of PC2 over PC1/3.

**Table 2:** Percent sequence identity and gaps between the mouse PC2 and template sequences.

	Overall	Catalytic domain^b^	P-domain^b^	Gaps
Furin (1P8J)^a^	52%	56% (109–444)	44% (445–578)	3%
Kex2 (1OT5)^a^	39%	44% (123–461)	29% (462–599)	4%

a The pdbids for each template are given.

b The residue numbering is for the intact pro-protein for the domains in the given templates.

### 6.) Searching for Alternative Binding Pockets

**Figure 7:**
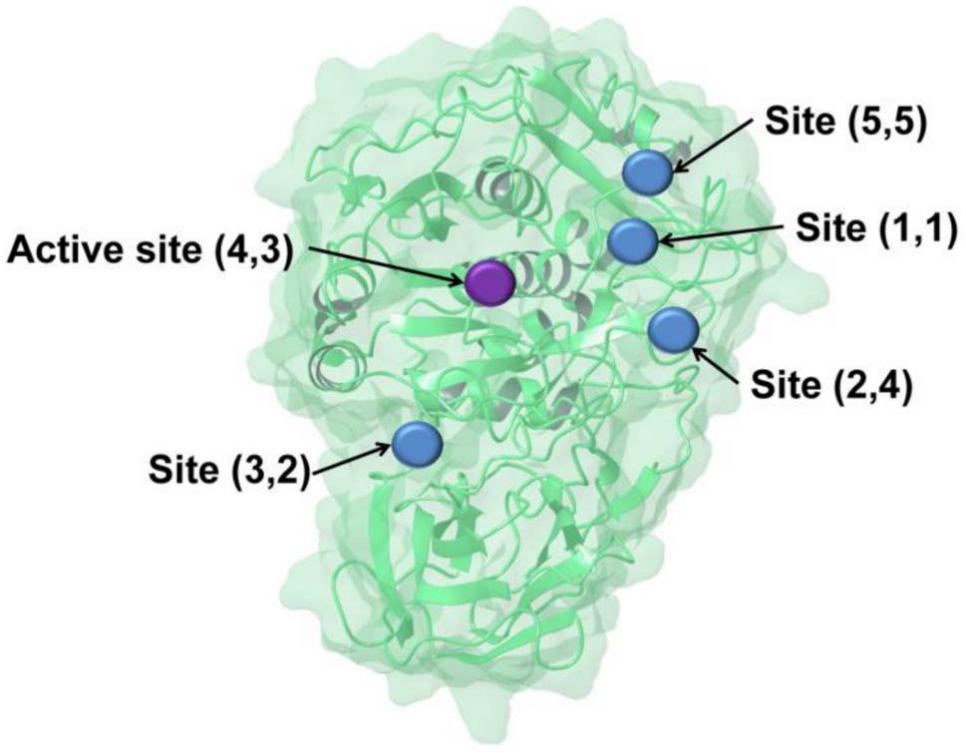
The active site and potential allosteric sites of PC2, as determined using two structural models of the enzyme. The first and second numbers in parentheses denote the ranking of each site in model 6 and the homology model, respectively. (See the section *Generating structural models of PC2 for ligand docking* for details).

The experimental results prompted us to evaluate computationally alternative binding sites. Docking scores were evaluated at putative allosteric binding sites. **Figure 7** shows putative allosteric binding sites for PC2 identified using Sitemap. The best score was for a proposed allosteric site, Site (1,1) close to one of the coordinated calcium ions. The binding score for RJC00847 at this putative allosteric binding site is −10.03 kcal/mol, whereas in the P1 pocket of the catalytic binding site the score is −8.23 kcal/mol.

## Discussion

In previous studies, peptides, peptidomimetics and non-peptidyl small molecules have been tested for potential inhibition of PC1/3 and PC2. Peptide and peptidomimetic inhibitors with Ki values in the 3.2–92 nM [22] and 0.81–78 nM [30] ranges, respectively, were identified for PC1/3. However, these inhibitors exhibited lower affinities towards PC2, with Ki values in the 360–2300 nM [22], [23] and 55–10000 nM [30] ranges. The PC1/3 pro-peptide has been shown to inhibit PC1/3 with a Ki value of 6 nM [20]. Other large peptide sequences, for example proSAAS, an endogenous PC1/3 inhibitor coexpressed with PC1/3, are also relatively weak, inhibiting PC1/3 with a Ki of 540 nM [27], while CRES was shown to selectively inhibit PC2 over PC1/3, with a Ki value of 25 nM [6]. The removal of the N-terminus of CRES resulted in lower affinity [6], indicating that this sequence is important for inhibition. In the area of non-peptidyl inhibitors, the first natural products demonstrated to have inhibitory activity towards PC1/3 were diterpine andrographolides [25] from *Andrographis paniculata* with Ki values in the high micromolar to millimolar range [31]. Interestingly, succinoylation of the andrographolides resulted in derivatives with Ki values in the low micromolar range towards PC1/3 and that were ineffective inhibitors of PC2 [31].

The involvement of PCs in a wide range of normal and pathological conditions highlights their importance as therapeutic targets. Selective inhibition of PC2 is a strategy to regulate glucagon production towards the management of diabetes. There are few PC2 inhibitors reported in the literature. Hence, the discovery of structurally diverse PC2 inhibitors will provide a wider repertoire of ligands with a variety of pharmacophoric and physicochemical properties.

From a kinetic perspective, ligand interactions with PCs are complex, displaying allosteric or orthosteric inhibition as well as stimulation [24]. With regard to this last feature, different forms of PC1/3 (66 kDa/74 kDa and 87 kDa) have been previously studied to investigate the influence of the C-terminal domain on stimulatory effects [9], [32]. We have previously observed a concentration-dependent type of inhibition for 2,5-dideoxystreptamine derivatives towards PC2. One of these compounds, 166369, inhibited PC2 as well as 87-kDa PC1/3 at high concentrations, but stimulated the 87-kDa form of PC1/3 at low concentrations. Meanwhile, compound 166369 inhibited the 66-kDa form of PC1/3 at both low and high concentrations [32]. The difference between these two forms of PC1/3 consists of the presence of an additional 21-kDa carboxyl-terminal domain in the 87-kDa form (see Vivoli *et al.* [32] and Cameron *et al.* [9] for details). We hypothesized that compound 166369 was capable of binding to at least two different sites in the protein, resulting in opposite effects: inhibition and stimulation. This hypothesis, along with the fact that a greater number of noncompetitive inhibitors have been identified than competitive PC2 inhibitors, should be considered when designing or searching for PC2 ligands. From a computational perspective, this offers an interesting perspective on convertase structure, possibly involving conformational changes of PC2 upon ligand binding at different binding sites.

In the work described here, we limited our docking studies into the active site. It should be mentioned that the fact that a compound binds in an allosteric site, as determined experimentally in a later stage: a) does not rule out the possibility of binding in the catalytic site, as in the case of concentration-dependent inhibition; and b) does not discredit docking studies, but rather highlights that for PC2, virtual screening based on molecular docking studies should include putative allosteric binding sites.

The selective PC2 inhibitor identified in this study is structurally different and of smaller size than those reported previously. This is advantageous for future studies where structural analogues can be built upon.

### Conclusions

Mouse PC1/3 and PC2 homology models have been developed and validated through molecular docking employing a set of decoys and/or known active compounds. Structure-based virtual screening of the Maybridge database identified potential PC2-selective compounds. The majority of the top-scoring compounds preferentially docked at the P4 subsite in the binding pocket of PC2. Re-docking of the top 115 PC2-ranked compounds employing FRED revealed that FRED ranked higher molecular weight compounds more favorably relative to GlideXP. Thus a consensus of the FRED and GlideXP was utilized to propose compounds for biological evaluation. Interestingly, fifteen of the top seventeen PC2-ranked compounds were not amongst the top 50 compounds ranked by PC1/3. Screening of these compounds revealed a non-competitive and selective PC2 inhibitor, RJC0084, with IC50 value of 1.1 µM. This is the first report of a small-molecule, non-peptide based PC2 inhibitor obtained with the aid of computational modeling.

## Methods

### 1). Generating a Homology Model of Mouse PC2

The furin crystal structure, reported at 2.6 Å resolution, is valuable for the development of furin inhibitors but also represents an excellent template to develop homology models of other PCs. For example, homology models of human PC1/3 and PC2 have been reported [7]. Here, we developed the homology model of the mouse PC2 enzyme (sp|P21661.1|NEC2_MOUSE). The target sequence was downloaded from the BLAST NCBI database. The homology modeling was performed using the Prime utility of Schrödinger, Inc. [42], which provides an interface for homologue searches, sequence alignments, template superposition, coordinate building and side chain optimizations. The crystal structures of two pro-protein convertases served as templates for the target sequence: furin (pdbid: 1P8J) [43] and Kex2 (pdbid: 1OT5) [44]. Prior to coordinate assignment, the structures of the two templates were superimposed. Furin was primarily employed, while coordinates were taken from the Kex2 structure when gaps or insertions were present in the aligned sequences of mouse PC2 and furin. In the course of generating the models, residues not derived from the templates as well as side chains were optimized. Finally, the stereochemical quality of the protein model was checked employing PROCHECK [45], accessed from the ExPASY server.

### 2). Generating Structural Models of PC2 for Ligand Docking

To account for receptor flexibility during ligand docking, an ensemble docking approach was employed. The different receptor conformations were generated by performing explicit-solvent molecular dynamics (MD) simulations initiated with the homology model. The par_all27_prot_lipid.inp CHARMM parameter file was employed to model the protein. The simulations were carried out employing NAMD v2.6 at Florida State University’s HPC center. Initially, two calcium ions important for the enzyme’s activity were included in the homology model. The first Ca^2+^ ion, close to the binding site, coordinated with the side chains of Asp161, Asp203, Glu233, and the backbone carbonyl group of Asp204; while the second Ca^2+^ coordinated with the side chains of Asp11, Asp64, Asn110, and the backbone carbonyl groups of Ala107, Ile112 and Gly114 to form an octahedral geometry. The enzyme was immersed in a box of 12026 TIP3P [46] water molecules and sodium ions were added to neutralize the system. The final dimensions of the system were 80×80×80. Initially, the positions of the water molecules and counter ions were optimized for 5000 steps employing the conjugate gradient (CG) method, while the enzyme and Ca^2+^ ions were constrained at their equilibrium positions with a 10-kcal/mol harmonic force constant. Next, the energy of the entire system was minimized for 5000 CG steps, while constraining the backbone atoms of the enzyme. A 50-ps heating stage ensued in which the temperature of the system was raised from 0–310 K in increments of 10 K/ps. This heating was followed by the 1-ns isothermal-isobaric production stage employing a 1.0-fs time step to integrate the equations of motion. Berensen’s pressure coupling method was utilized to maintain a target temperature of 1.01 atmospheres. In the course of the heating and production stages, the enzyme’s backbone atoms were constrained with a 10-kcal/mol harmonic force constant. The particle-mesh Ewald method [47] was employed to compute long-range electrostatics in both the equilibration and production stages. The non-bonded cutoff, pair-list distance, and pair-lists per cycle were 12.0 Å, 14.0 Å, and two, respectively, while the switch distance, margin and steps per cycle were 8.0 Å, 2.5 Å and 20, respectively. Coordinates from the trajectory were taken every 1 ps.

To select enzyme structures for docking, the trajectory was clustered based on root-mean-squared deviation (RMSD) utilizing the gromos method of the g_cluster trajectory analysis tool in GROMACS [48]. First, the frames of the trajectory were superimposed onto the first frame using backbone atoms in order to remove any rotational or translational degrees of freedom. Given that the backbone atoms of the enzyme were constrained with 10-kcal/mol harmonic force constant in the course of the heating and production stages of the MD simulation, no differences were observed in the overall folds of the models used for docking. Thus we analyzed side chains within a 10-Å radius of the binding pocket, paying particular attention to those that were solvent exposed. Notable side chain differences between the homology model and model6 from the MD simulation were observed in the binding pocket. Comparisons of the overall fold and side chain differences of solvent exposed residues in the binding pocket are shown in **Figure S5**. The overlaid structures were then clustered using residues within 10 Å of the binding pocket with a 0.75-Å RMSD cutoff. Centroids from the top five most populated clusters were selected. An additional structure (the centroid of the sixth most populated cluster) was also included because the solvent-accessible surface area [49] of the residues within 10 Å of its binding pocket was remarkably different from those of the other centroids. These models are denoted: *Homology*; and *model1*, *model2*, *model3*, *model4*, *model5* and *model6* derived from the MD simulation.

### 3). Ligands and Decoys

Eleven ligands with known activities towards PC2 were selected for docking. These comprised seven peptides and four pyrrolidine bis-piperazine small molecules, shown in **Table 3**. While the peptides bind in a competitive manner [22], [23], the small molecules were identified as non-competitive inhibitors [24]. All the ligands were included in the study, with the aim of spanning different types of ligands. For each available active ligand 20 decoys were selected from the directory of useful decoys (DUD) database using a “decoy score” based on physico-chemical (PK) property mimetics and modeled 3D shape comparisons [50]. The workflow for decoy selection is presented as supplementary material, **Figure S1**. Initially, the Molecular Operating Environment [51] interface was employed to compute seven drug-like PK properties: hydrogen bond acceptors/donors, logS, SlogP, molecular weight, number of rotatable bonds and topological surface area (TPSA) for the actives as well as the decoys. Next, differences between the PK properties of each active and the decoys were computed (ΔPK). For the modeled component of the “decoy score” 3D structural similarities between each active and the decoys were determined employing the comboscore measure from the shape-based Rapid Overlay of Chemical Structures (ROCS) [52] module. Given that small a ΔPK indicated high PK similarity, while high magnitude comboscores (maximum value of 2) denoted high structural similarity, the absolute value of the complement comboscore [abs(comboscore –2)] was determined. After this procedure both small ΔPK and abs(comboscore –2) indicated good similarity with an active. The ΔPK and abs(comboscore –2) values were summed for each active, ranked in ascending order and the top 20 decoys were selected for docking. These decoys were removed from the database and the process of computing the “decoy score” was iterated for all the other actives.

**Table 3:** Maybridge Compound Screening of PC1/3 and PC2.

	% inhibition							
	PC1/3				PC2			
Compound	50 µM		25 µM		50 µM		25 µM	
**RJC00847**	6	(1.2)	10	(2.8)	**98**	**(0.2)**	**86**	**(0.1)**
**BTB03195**	**−41**	**(3.6)**	**−6.5**	**(0.2)**	**71**	**(0.7)**	**48**	**(25.9)**
CD04301	12	(2.6)	13	(3.0)	44	(6.8)	9	(5.3)
HTS04864	12.5	(1.1)	9	(3.6)	41	(7.9)	17	(0.7)
SCR01261	12	(10.5)	7	(1.6)	38	(7.1)	43	(1.7)
HTS09720	2.5	(10.5)	3	(2.8)	33	(3.1)	8	(21.2)
RJC03465	0.6	(0.3)	6	(5.7)	28	(8.6)	38	(2.5)
RH01866	6	(0.2)	11	(4.8)	24	(10.4)	36	(1.5)
CD10677	−0.2	(8.8)	1.5	(1.3)	11	(7.2)	−29	(46.4)
CD03118	13	(5.4)	12	(0.9)	5	(10.2)	39	(1.2)
RH02062	3	(4.2)	6	(0.0)	4	(1.3)	−1	(9.9)
**JFD02062**	15	(0.6)	13	(3.2)	**−60**	**(8.3)**	**−30**	**(0.9)**
**HTS05737**	8	(1.1)	12	(1.1)	**−282**	**(8.0)**	4	(4.9)

Negative numbers represent stimulation.

% error is shown in parenthesis.

Bold represent compounds with relevant inhibition or stimulation effect towards either PC.

### 4). Docking

The seven enzyme structures and 231 ligands (11 actives and 220 decoys) were prepared employing the protein preparation wizard and ligPrep [53] modules of Schrödinger, Inc., respectively. The dimensions of the inner and outer grids were 14×14×14 and 50×50×50, respectively. Ensemble docking was performed utilizing the virtual screening workflow (vsw) module and the GlideXP scoring function.

To compare the effectiveness of each model in retrieving active compounds from the database, the fraction of actives retrieved as a function of the fraction of the entire database screened was determined. These values were plotted and the area under each curve (AUC) was determined employing Origin v8 [54]. Higher AUC values indicate better performance. Based on these initial rankings two ensembles were created by grouping *Homology*, *model3* and *model6* in one set called *Hom_mod3_mod6*, and *Homology* and *model6* in another set, named *Hom_mod6*. The aim was to utilize as few receptor models as possible, while retrieving a high percentage of ligands. The ensemble that produced the best recovery was employed to dock 14,400 compounds from the Maybridge database.

### 5). Biological Assays

Recombinant PC2 was assayed in the presence and absence of inhibitors using the standard fluorogenic substrate, pERTKR-aminomethyl coumarin, as previously described [24], [32]. Inhibitors were preincubated with enzyme for 30 min prior to addition of substrate.

For the IC_50_ assay, the compound RJC00847 was placed into 96 well-plate containing PC2 and his corresponding buffer [21], [29]. Serial dilutions of compound RJC00847 were performed to give final concentrations between 10 nM to 500 µM in 50 µl. After a 30 min preincubation at room temperature, pERTKR-amc (100 µM final concentration) was added and residual enzyme activity was monitored by measuring the emission of amc fluorescence. Data were analyzed using GraphPad Prism 5 (GraphPad Inc., San Diego, CA). The sigmoidal curve obtained was fitted with the following equation:
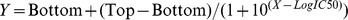



Top and Bottom are plateaus in the units of the Y axis, which represents the rate, expressed as RFU/min; X represents the logarithm of substrate concentration. IC_50_ is the concentration of agonist that gives a response half way between Bottom and Top.

The study of PC2 inhibition kinetics was performed at various concentrations of pERTKR-amc ranging from 0 to 200 µM in the presence and in the absence of compound RJC00847. Prior to the addition of substrate an 30 min preincubation oof PC2 with the compound was carried out. The assay was performed in duplicate in 96-well microplates. The data were analyzed using GraphPad Prism 5 and the inhibition constant was determined using the following equation, describing an uncompetitive mechanism of inhibition for the tested compound:
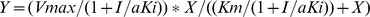


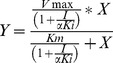
where Vmax is the maximum enzyme velocity without inhibitor, expressed in the same units as Y (RFU/min); Km is the Michaelis-Menten constant (without inhibitor), expressed in the same units as X, corresponding to the concentration of substrate and αKi is the inhibition constant, expressed in the same units as I, which represents the concentration of the inhibitor.

### 6.) Searching for Alternative Binding Pockets

Possible binding pockets for PC2 were determined using the sitemap program in Maestro, employing the two models that we used for virtual screening (Homology model and model6). The top 5 scoring sites (based on the number of site points, hydrophobic, hydrophilic, hydrogen bond acceptor, donor and metal-binding maps) of each model including the active site were selected. These putative binding sites were used for docking studies. Proposed binding sites located adjacent to each other by visual inspection were enclosed in one grid box. This grouping resulted in two grids for the homology model (AlloActSite_23, AlloSite_145) and three grids for model6 (ActSite_4, AlloSite_3, AlloSite_125). Act = active site; Allo = allosteric site; AlloAct = grid enclosed both an allosteric and the active site; the numbers are derived from the output of sitemap. RJC00847 was docked to these sites using the virtual screening workflow tool of Maestro and the scores were merged to identify best scoring site.

## Supporting Information

Figure S1 Workflow employed to select decoys from the Directory of Useful Decoys database (DUD). The physico-chemical properties (PK) were: hydrogen bond acceptors/donors, logS, SlogP, molecular weight, number of rotatable bonds and topological polar surface area.
(TIFF)

Figure S2 Superposition and alignments of mouse PC2, furin (1P8J) and Kex2 (1OT5) structures and sequences, respectively. In the superimposed representation the homology model, furin and Kex2 are shown in blue, cyan and red, respectively. The Cartesian coordinates of the residues from each template highlighted in red were employed to generate those of the homology model. The coordinates of the residues highlighted in cyan were generated entirely using rotamer libraries in Prime.
(TIFF)

Figure S3 Area under recovery curves as a function of the percent of the database screened for the seven models employed to dock the actives and decoys dataset. The numerical values at 10% of the database screened are shown on the right.
(TIFF)

Figure S4 Property space overlap between selected PC2 actives and decoys from the DUD database.
(TIFF)

Figure S5 (**A**) Overlay of the backbone atoms of the models employed to perform ensemble docking. Homology model: magenta; model6: cyan. There are no differences in their overall folds (backbone RMSD = 0.16 Å) given the 10-kcal/mol backbone constraint employed in generating the models during molecular dynamics simulations. (**B**) Side chain differences between the homology model (magenta) and model 6 (colored by atom type) from the MD simulation.
(TIFF)

Table S1 Plot statistics for non-glycine and non-proline residues.
(TIFF)
