# Supplementary figures and images for: Identification of a Small Molecule That Selectively Inhibits Mouse PC2 over Mouse PC1/3: A Computational and Experimental Study
Source: PLoS One. 2013 Feb 22;8(2):e56957. doi: 10.1371/journal.pone.0056957 (PMC3579927; doi:10.1371/journal.pone.0056957)

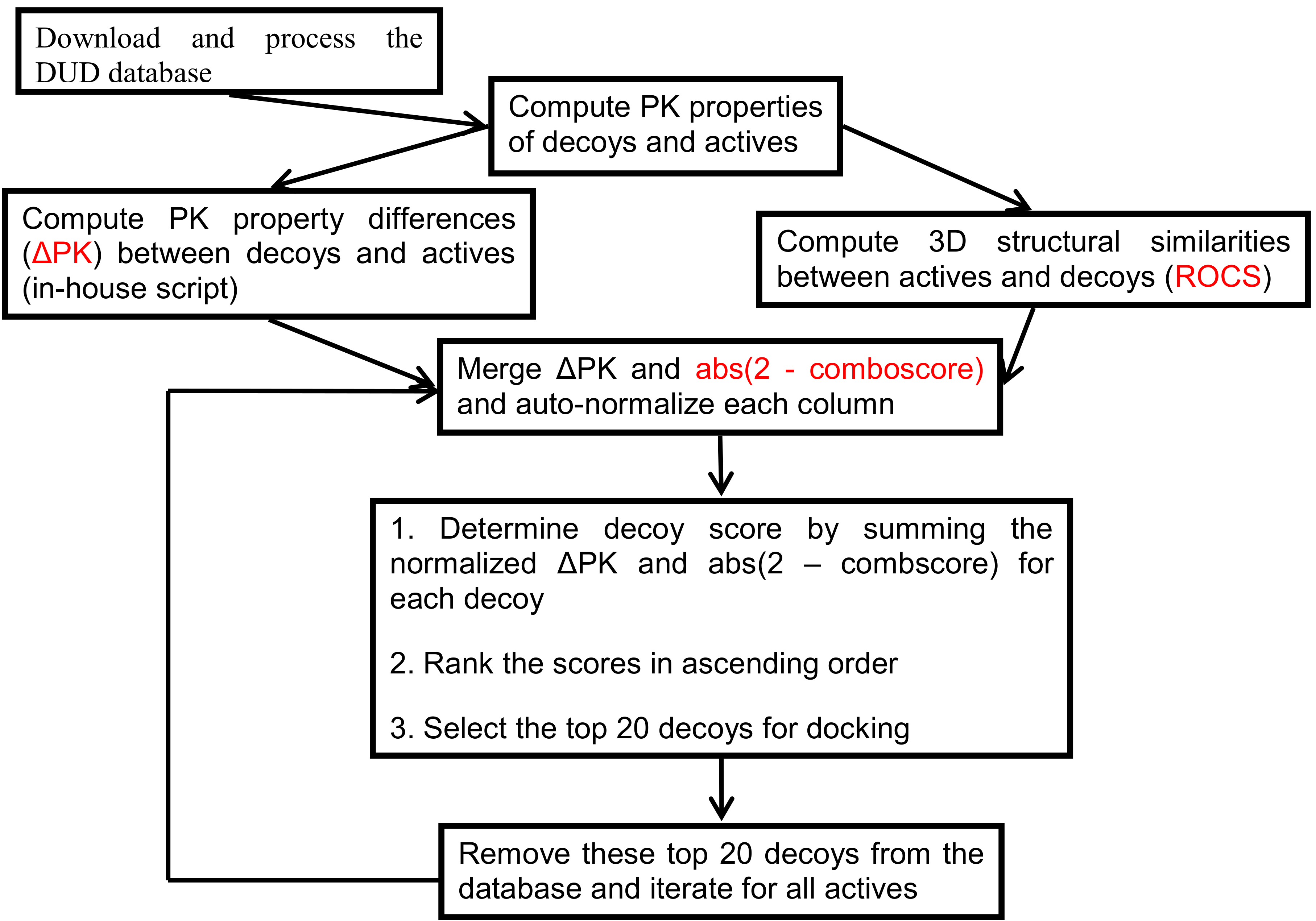

Supplement: Figure S1 — Workflow employed to select decoys from the Directory of Useful Decoys database (DUD). The physico-chemical properties (PK) were: hydrogen bond acceptors/donors, logS, SlogP, molecular weight, number of rotatable bonds and topological polar surface area. (TIFF) [file pone.0056957.s001.tiff]

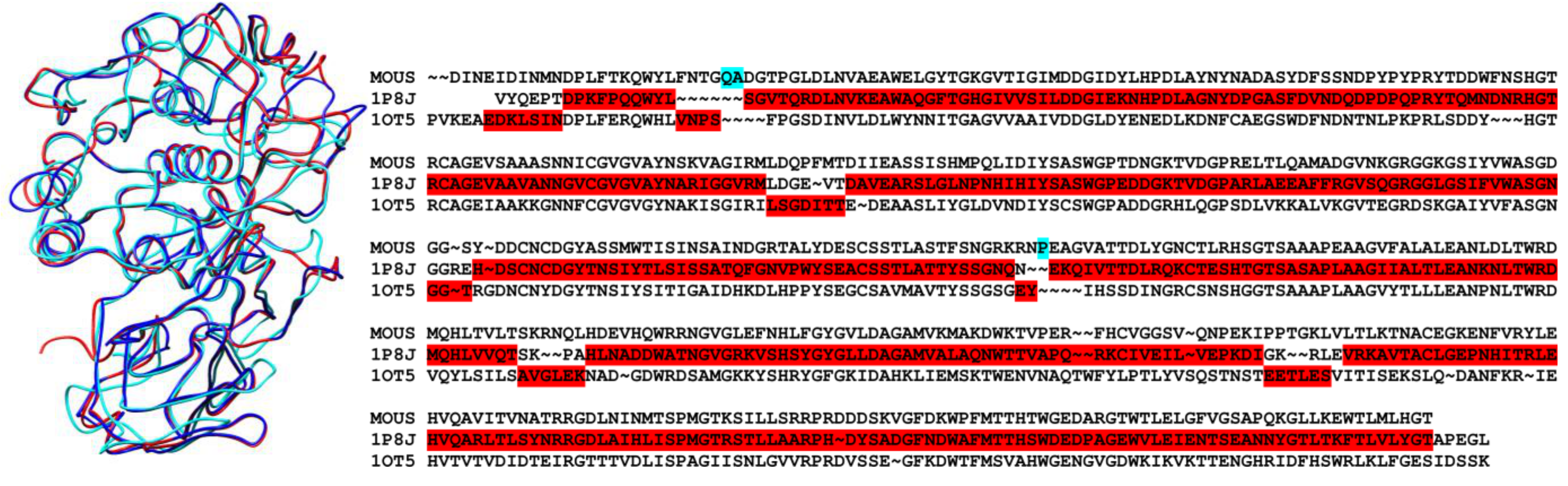

Supplement: Figure S2 — Superposition and alignments of mouse PC2, furin (1P8J) and Kex2 (1OT5) structures and sequences, respectively. In the superimposed representation the homology model, furin and Kex2 are shown in blue, cyan and red, respectively. The Cartesian coordinates of the residues from each template highlighted in red were employed to generate those of the homology model. The coordinates of the residues highlighted in cyan were generated entirely using rotamer libraries in Prime. (TIFF) [file pone.0056957.s002.tiff]

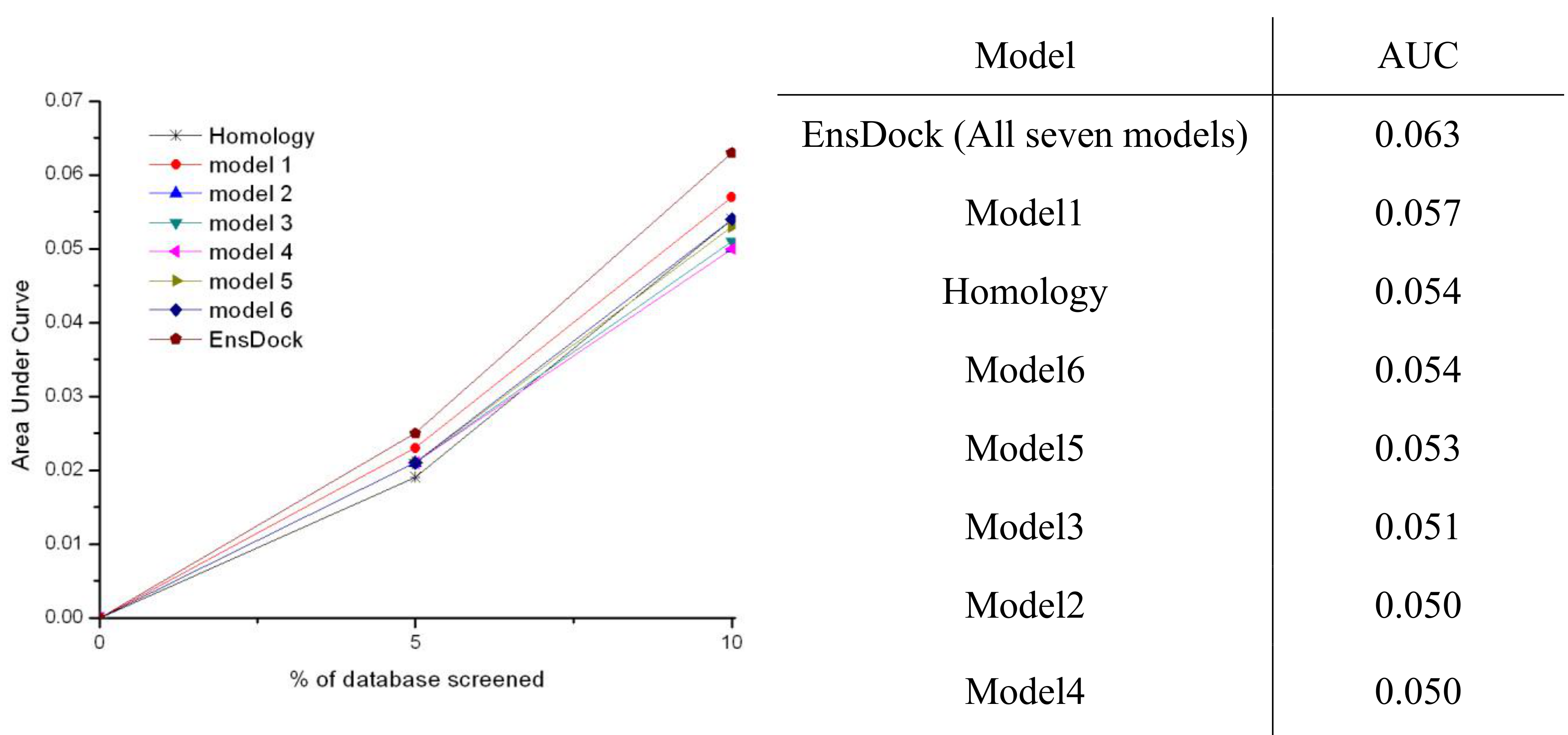

Supplement: Figure S3 — Area under recovery curves as a function of the percent of the database screened for the seven models employed to dock the actives and decoys dataset. The numerical values at 10% of the database screened are shown on the right. (TIFF) [file pone.0056957.s003.tiff]

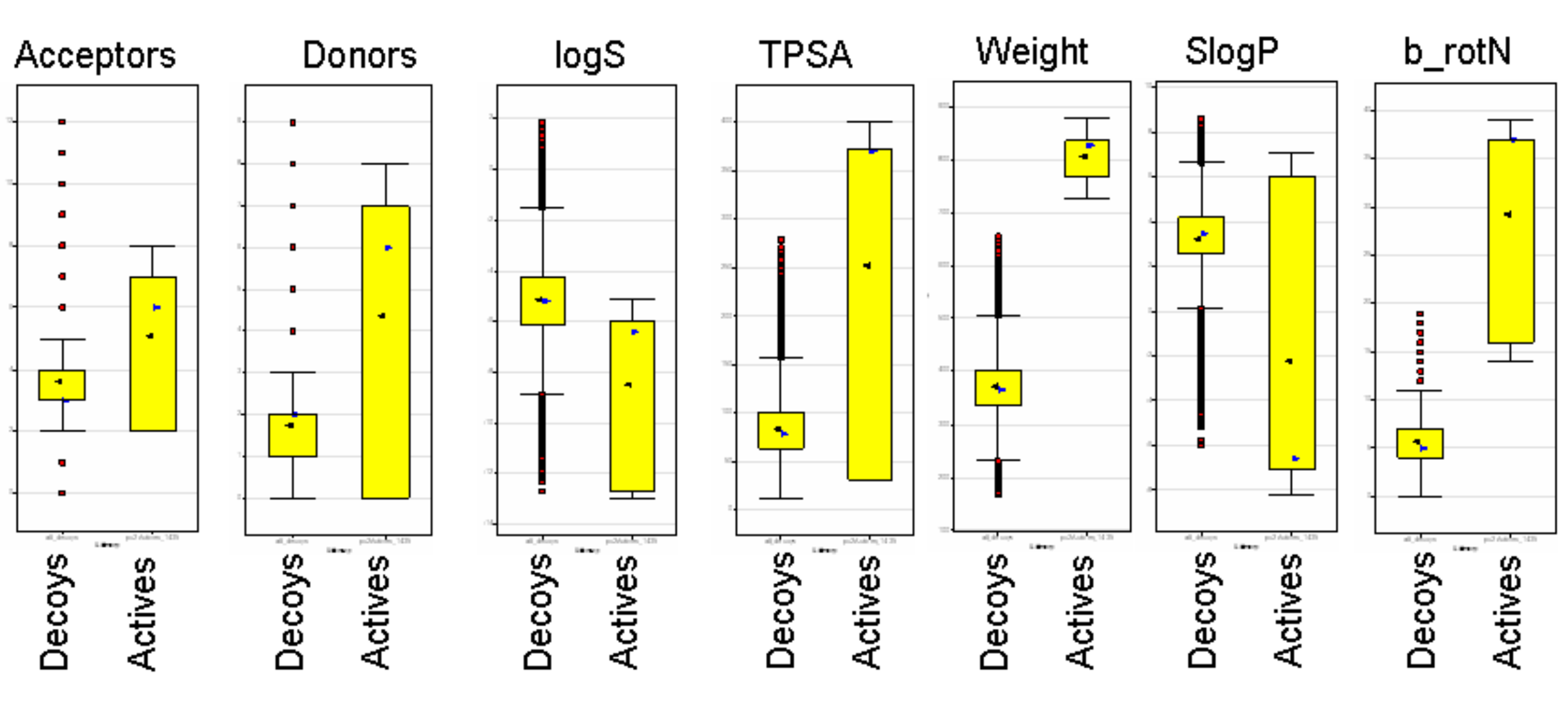

Supplement: Figure S4 — Property space overlap between selected PC2 actives and decoys from the DUD database. (TIFF) [file pone.0056957.s004.tiff]

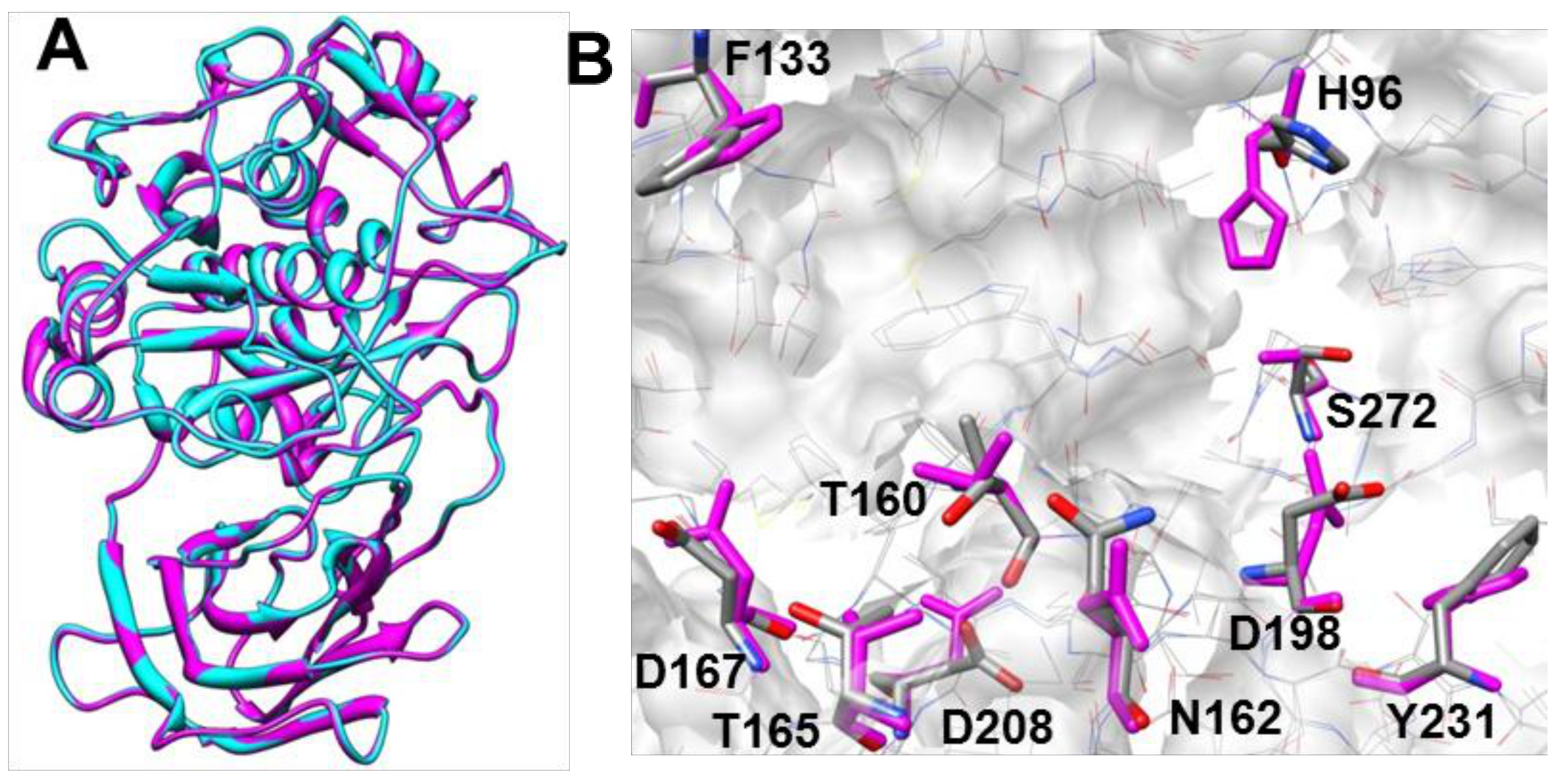

Supplement: Figure S5 — (A) Overlay of the backbone atoms of the models employed to perform ensemble docking. Homology model: magenta; model6: cyan. There are no differences in their overall folds (backbone RMSD = 0.16 Å) given the 10-kcal/mol backbone constraint employed in generating the models during molecular dynamics simulations. (B) Side chain differences between the homology model (magenta) and model 6 (colored by atom type) from the MD simulation. (TIFF) [file pone.0056957.s005.tiff]
